# A Systematic Review of the Sedative, Behavioral, Analgesic and Cardiovascular Effects of Gabapentin in Cats

**DOI:** 10.3390/vetsci12100938

**Published:** 2025-09-28

**Authors:** Marianna Virginia Laguardia, Martina Polvere, Claudia Piemontese, Floriana Gernone, Francesco Staffieri

**Affiliations:** 1 Department of Veterinary Medicine, SCICOV, University of Bari “Aldo Moro”, 70010 Bari, Italy; m.polvere@phd.uniba.it; 2 PhD Course in Organs and Tissues Transplantation and Cellular Therapies, DiMePreJ, University of Bari “Aldo Moro”, 70010 Bari, Italy; c.piemontese1@phd.uniba.it; 3 Department of Veterinary Medicine, University of Bari, 70010 Valenzano, Italy; floriana.gernone@uniba.it; 4 Section of Veterinary Clinics and Animal Production, DiMePreJ, University of Bari “Aldo Moro”, 70010 Bari, Italy; francesco.staffieri@uniba.it

**Keywords:** gabapentin, felines, sedation, behavior, pain management

## Abstract

**Simple Summary:**

Gabapentin is popular in veterinary medicine due to its depressive effects on the central nervous system, analgesic properties, and behavioral impact. Stress or aggressive behavior can make handling cats during clinical examinations extremely difficult. This systematic review, based on twenty selected articles, aims to evaluate gabapentin’s impact on sedation, anxiety, behavioral modification, pain, and cardiovascular function in feline patients during veterinary appointments. The review shows that gabapentin mildly to moderately reduces anxiety in cats. However, responsiveness may vary depending on the dosage used. Gabapentin can be orally administered mixed with food and does not negatively impact the cardiovascular system. Overall, gabapentin may reduce stress in cats during veterinary examinations.

**Abstract:**

Gabapentin is a drug frequently used in veterinary medicine because of its recognized analgesic, sedative, and behavioral properties. In recent years, its use has become particularly important in feline medicine. The clinical examination of a cat can be challenging due to various factors, such as patient compliance or inadequate handling techniques employed by veterinary staff, which can lead to fear-based aggressive behavior in cats. A systematic review based on the PRISMA statement was conducted from March to September 2024. Out of 543 articles, only 20 were included in the review. The objective of the systematic review was to describe the impact of gabapentin on sedation, anxiety, behavioral modification, pain, and cardiovascular function in feline patients during veterinary appointments. Gabapentin’s effects may be dose-dependent, though a specific dosage is not available. Administering gabapentin with wet or dry food is suggested. Furthermore, gabapentin has positive behavioral, analgesic, and sedative effects, ensuring an anxiolytic effect without altering any cardiovascular, echocardiographic, or hemodynamic aspects.

## 1. Introduction

Gabapentin (1-aminomethylcyclohexane acetic acid) is a drug that functions as an analogue of gamma amino butyric acid (GABA) without altering the binding, re-uptaking, or degradation of this neurotransmitter. It seems to exert its action by binding the alpha 2 delta-1 subunit of voltage-gated calcium GABA-associated receptors within the central nervous system [1]. By reducing calcium influx, it inhibits the release of excitatory neurotransmitters (e.g., substance P, glutamate, norepinephrine) [2]. Morphologically, it appears as a crystalline, hydrophilic substance with a bitter taste [3].

The optimal method of administration for gabapentin in cats is via the oral route. This is due to the lower bioavailability of transdermal administration [1].

The absorption rate is about 3 h after the ingestion in most species. In cats, it has a bioavailability average of 90% after oral administration, but a considerable degree of variation has been observed among patients. It was determined that peak levels in cats occurred approximately 100 min after the administration of the drug [4]. The volume of distribution is relatively low (Vdss of 0.65 L/kg). It is excreted by the kidney and entirely eliminated through the urine [5]. The clearance in cats is about 3 mL/min/kg, with a mean elimination half-life of 2.8 h [6]. This drug was first introduced in human medicine approximately 40 years ago to treat neuropathic pain and refractory partial seizures, with rare reported side effects [7]. In cats, gabapentin was used off-label at first as an anti-epileptic drug [8]. Nowadays, it is prevalently used to manage chronic and neuropathic pain [9]. Additionally, in cats, gabapentin showed efficacy in anxiety management [10].

Moreover, regarding its use as an analgesic in neuropathic conditions, this pharmaceutical agent has been demonstrated to be efficacious in the veterinary management of pain associated with spinal cord injury and similar disorders [2].

In recent years, gabapentin has also been used extensively in veterinary behavioral medicine with the aim of reducing stress levels in cats by making them more compliant during physical evaluation [11].

It is important to emphasize that this is still an off-label drug and its evidence in veterinary medicine is limited for neuropathic pain conditions other than behavioral and sedative effects.

Stress in human medicine is defined as a condition in which an environmental demand exceeds the body’s ability to regulate itself, especially when this event is unpredictable and uncontrollable [11]. In veterinary medicine, the term “stress” is a multifaceted concept with numerous definitions. From the perspective of health and disease, stress is frequently defined in terms of stressor. Therefore, a stressor could be any agent that instigates the activation of the central threat response system [12]. Buffington [13] proposed a taxonomy of feline stress duration, grounded in Shonkoff’s classification of stressor potential and response duration [12]. It was divided into moderate, mild, and toxic. Mild stress is context-dependent and may facilitate autoregulation during new situations. These reactions to stress occur within the safe, predictable environment of stable and supportive relationships (e.g., exposing the kitten to new stimuli at home). Moderate stress arises when threat is greater than the mild one, such as boredom or an acute illness. Unlike mild stress, the cat is exposed to moderate stress for a longer period but is able to regulate itself because its surroundings allow it to calm down. Finally, toxic stress, more known as chronic stress, is the most threatening one as it may not be possible for the cat to return to its basal state. In fact, some situations, such as mistreatment of the animal, chronic illness, a poorly performed physical evaluation, or a prolonged hospital stay, can lead to chronic stress.

In fact, the relationship between cats and veterinarians during the clinical examination is well known to be complicated and sometimes challenging, as being in a new environment combined with unfamiliar stimuli and smells causes the animal to experience high levels of stress. Therefore, cats show significantly lower stress values when examined at home compared to a hospital environment [14]. Furthermore, cats are not used to the restraint and manipulation required for the physical examination. Indeed, all of this could lead the cat to react by following the fight-or-flight behavioral pattern, attempting to flee or attack the operator. Furthermore, an inappropriate approach or inexperience could be responsible for anxiety or phobia on subsequent visits. All these events could lead to the development of avoidance or aggressive behavior, even towards the owner, prior to future veterinary appointments. In addition, the cat may experience discomfort from being placed in the carrier on the way to the waiting room and, finally, to the clinical examination table. This can significantly alter not only the cat’s behaviours but also clinical parameters and consequently diagnosis and treatment, not to mention the physical danger to the operator when dealing with these subjects [15]. To improve this issue, behavioral medicine can be employed. Unfortunately, appropriate cat handling is not often applied by veterinary staff and owners in most facilities, and this represents a threat to the welfare of the animal [16]. Consequently, euthanasia may be used when the animal’s behavior is deemed to be untreatable [17]. In order to improve the relationship between veterinarians and cats, there are both practical and pharmacological alternatives that help the animal to greatly reduce stress levels and, consequently, ensure a much more positive experience in unfamiliar situations. On one hand, it is possible to adopt manual practices such as avoiding scruffling the cat, having a kinder and more patient approach to it, and environmental enrichment of the living areas [18]. On the other hand, there are number of drugs on the market that aim significantly reduce stress levels and improve interaction with humans and veterinarians such as Alpha-casozepine [18], Trazodone, and gabapentin [19]. Due to the limited available bibliography and to standardize the data related to gabapentin, this review focuses exclusively on the latter drug.

This systematic review may provide clearer indication to the practitioner on the use of gabapentin in cats considering the different clinical needs. The aim of this review is to describe the impact of gabapentin on sedation, anxiety, behavioral modification, pain, and cardiovascular function in feline patients during veterinary appointments.

## 2. Materials and Methods

A systematic literature search was conducted based on the Preferred Reporting Items of Systematic reviews and Meta-Analysis, PRISMA 2020 statement [20]. Eligibility criteria were the following:

- Population: Domestic cats (*Felis silvestris catus*), regardless of breed, sex, or health status.
- Intervention: Oral administration of gabapentin at any dosage and frequency.
- Comparator: Placebo, no treatment, or other sedative, anxiolytic, or analgesic agents.
- Outcomes: Defined a priori as: behavioral changes; sedative effects; analgesic effects; cardiovascular responses; adverse effects.
- Study design: Randomized controlled trials (RCTs), non-randomized controlled trials, observational studies, case reports, systematic reviews, and meta-analyses. Editorials and abstracts were excluded.

The search strategy was submitted on three main databases: Pubmed, Web of Science, and Scopus, between 1 March 2024 and 30 September 2024. The keywords used for the research were “gabapentin”; “cats OR feline” using the Boolean operator “AND”, limited to studies published in Italian, English, Spanish, or French. Titles and abstracts were screened by two of the authors independently. Articles not related to the analgesic, behavioral, or sedative effect of gabapentin in cats were ruled out. Articles which administered gabapentin to cats via a route other than oral were ruled out. Articles that used gabapentin in combination with other drugs were also excluded (such as multimodal anesthesia). Articles that evaluated the single effect of gabapentin with other drugs with similar effects such as Alprazolam or Melatonin were included. Primary outcomes included sedative and behavioral effects. Secondary outcomes were cardiovascular and analgesic effects as well as adverse events. These outcomes were pre-defined prior to data collection. All studies published up to September 2024 were considered. No lower time limit was applied.

Risk of bias was assessed for each included study. For randomized controlled trials, the SYRCLE’s risk of bias tool was used [21]. For non-randomized studies, the ROBINS-I tool was applied [22]. Each study was evaluated independently by two reviewers with disagreement resolved by consensus.

## 3. Results

**Figure 1:**
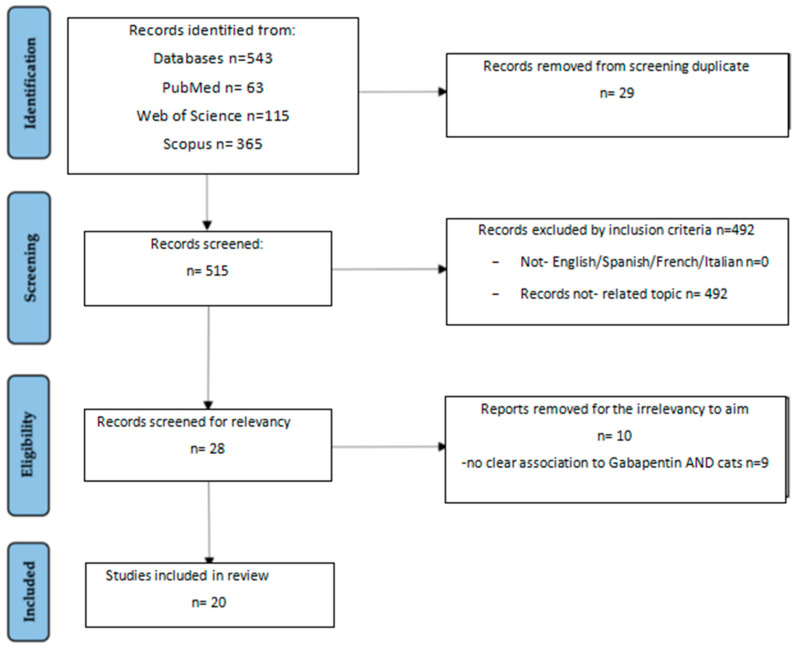
Study selection based on PRISMA flow chart.

The initial literature research from the three databases pointed out a total of 543 records: 63 from Pubmed; 115 from Web of Science, and 365 from Scopus. After removal of duplicates (n), 515 records were screened. Out of these 515 records, 497 were removed because they were not related to the topic, such as those examining multimodal association of gabapentin with other drugs or gabapentin used for other purposes, such as anti-epileptic use. In the end, a total of 20 articles were considered eligible for this review and were included (Figure 1)

The 20 articles assessed in this review are reported by specific criteria (see Table 1).

The included studies comprised six RCTs, assessed using the SYRCLE risk of bias tool, and thirteen observational studies or non-randomized controlled studies, assessed using ROBINS-I. One narrative review was excluded from bias assessment. Study purpose varied, focusing on sedative effects, behavioral responses, analgesia, cardiovascular safety, and side effects. Sample sizes ranged from 3 to 75 cats. Gabapentin dosages ranged from 9 to 47 mg/Kg, typically administered orally 60–120 min before assessment. Among the RCTs, four were judged to have low risk of bias, while two had moderate concerns. Overall, the methodological quality of RCTs was high with adequate randomization and outcome assessment. The non-randomized studies generally exhibited moderate to serious risk of bias, particularly due to confounding, lack of control groups, and subjectivity in behavioral outcome assessment. Three studies were considered at serious risk of bias due to lack of comparator and retrospective design. A complete risk of bias is provided in Table 2.

## 4. Discussions

This systematic review identified 20 articles focusing on the sedative, analgesic, behavioral, and cardiovascular effects, as well as the adverse effects, in cats. To the authors’ knowledge, no other systematic reviews on this topic were found in the databases used for the search (PubMed, Web of Science, and Scopus).

All RCTs reported that oral gabapentin significantly reduced stress levels and improved handling compliance, especially when administered 90–120 min before evaluation. The effect was dose-dependent in some studies [30], with enhanced side effects at ≥30 mg/kg [23]. Conversely, Kruszka [24] states that behavioral changes depend on the individual subjects and are not related to the dosage. Furthermore, the article emphasized a much calmer attitude when gabapentin was administered prior to transporting the cat from its familiar environment to the clinic. Behavioral scoring tools included the Cat Stress Score, Glasgow Composite Pain Scale, and Feline Grimace Scale. Several observational studies supported these findings, although individual variability was noted [24,36,37,40]. One article found that many cats classified as “fear-aggressive behavior” that were given a standard dosage of the drug [24] showed a noticeable behavioral change two hours after administration. The examined animals were much friendlier and more approachable toward the operator, with or without food present.

Four studies documented analgesic effects. Steagall [40] observed reduced postoperative pain following ovariohysterectomy when gabapentin was administered with buprenorphine. In an acute experimental model, Pypendop [23] found no effect of gabapentin on thermal antinociception. Lorenz [28] also reported that chronic pain decreased according to the animals’ activity after about one month of gabapentin administration in three cats with bone trauma. Guedes [38] reported improved pain scores in osteoarthritic cats after chronic administration. However, gabapentin alone did not demonstrate strong analgesic efficacy in the short term. Guedes [38] hypothesizes that, to observe a significant reduction in pain, gabapentin must be administered over a long period, emphasizing the role of the accumulation effect.

Four studies investigated cardiovascular parameters. Gabapentin did not significantly alter blood pressure, heart rate, or echocardiographic findings in healthy or hyperthyroid cats. One study reported a minor decrease in systolic function and T-wave polarity change, but these findings were not clinically significant [27,30,32,33]. However, it is possible to say that three of four studies had a moderate risk of bias; thus, the results may not be reliable.

Compared to placebo, gabapentin has consistent anxiolytic and sedative effects supported by behavioral and, in some cases, physiological data. Indeed, in almost all studies in which gabapentin was compared to placebo, it showed a great efficacy in reducing stress, improving compliance and promoting calmer behavior during the veterinary examination [25,29,39]. Papageorgiou [40] found that gabapentin and alprazolam produced comparable levels of sedation and anxiolysis. No significant differences were observed in post-operative outcomes, and post-operative profiles were similar. Thus, gabapentin offers a non-benzodiazepine alternative with similar efficacy but potentially safer long-term profile. Tuleski [30] demonstrated that gabapentin and melatonin significantly improved compliance during examination without affecting cardiovascular parameters. Comparative studies with Mirtazapine, Fantinatu [9], and Spano [41] found that both drugs increased appetite and improved demeanor, but gabapentin has a more direct impact on compliance and sedation. The combination of topical mirtazapine and oral gabapentin was synergistic, facilitating ingestion and enhancing calming effects.

According to some studies [24,25,29,31,32,39], side effects have been reported during gabapentin administration. The most common were ataxia, salivation, and stupor. These were often accompanied by protrusion of the third eyelid, fasciculations, vomiting, and diarrhea. Van Haaften [29] correlated these side effects with the dosage administered. Indeed, a higher dosage was associated with a greater likelihood of adverse effects emerging. Therefore, we can assume that the observed side effects are related to toxicity (dose-related or individual reaction), which resolved within eight to ten hours after administration [29]. Furthermore, no side effects were reported in cats with hyperthyroidism associated with gabapentin administration.

Outside the scope of this systematic review, we can make some considerations regarding dosages, intervals, and administration methods. There is no uniformity in dosing intervals in the reviewed literature. In fact, gabapentin administration schedules of once, twice, or three times a day were described. Furthermore, the administered dose of gabapentin varies. In most articles, the administered dose was 100 mg per cat, or 150 mg if the cat weighed more than 7 kg. Other articles administered a higher dosage of gabapentin than 150 mg per cat, such as 200 mg per cat or up to 30 mg/kg [23]. Only one article established the dose prior to the start of the study at 10 mg/kg [38]. The modality of gabapentin administration described in all the analyzed articles is uniform. It is, in fact, dispensed by the oral route, either via a mixture of wet or dry food [27] or providing it as it is [26]. Gabapentin was offered to patients in different ways: by offering the animal a bowl and assessing its intake capacity, by syringe with a Tom Cat catheter, or by combining it with wet food. Only one article [28] describes administering gabapentin in syrup form (gabapentin syrup 40 mg/mL, Nova Laboratories), which is an oil-based compounded liquid suspension. A case series reported the parenteral administration of gabapentin via a feeding tube [28].

## 5. Conclusions

This systematic review demonstrates that gabapentin has consistent and beneficial effects on key clinical outcomes in domestic cats undergoing veterinary procedures. Across the selected studies, gabapentin was shown to reliably induce sedation, reduce stress-related behaviors, and exert analgesic effects in both acute and chronic settings.

Behavioral improvements were particularly notable in fearful or aggressive cats, with increased compliance and reduced signs of anxiety during clinical examinations. Sedative effects were observed in the majority of studies, with clear facilitation of handling and physical evaluation. However, it is important to underline how side effects such as ataxia or sialorrhea may result as the dosage increases or due to individual reactions. In addition, gabapentin contributed to pain reduction, particularly in postoperative scenarios and chronic pain conditions, as evidenced by behavioral scoring systems and, in some cases, physiological indicators such as serum cortisol levels.

Importantly, gabapentin was well tolerated across the included studies. Reported adverse effects were generally mild and transient, with no significant alterations in cardiovascular or echocardiographic parameters.

In conclusion, gabapentin emerges as a multifunctional pharmacological option in feline veterinary medicine, providing consistent sedation, behavioral calming, and analgesia. These properties make it a valuable tool for enhancing feline welfare during clinical procedures. Further research is encouraged to support the development of standardized dosage and outcome measures and expand evidence on its long-term safety and efficacy.

## Figures and Tables

**Table 1:** Summary of the 20 selected studies, including author, year of publication, number of cats enrolled, gabapentin dosage, purpose of the study, study outcomes, number of references cited, article type, reported effects related to gabapentin administration, and scoring systems used.

**Articles**	**Appetite Stimulating Effect of Gabapentin vs. Mirtazapine in Healthy Cats Post-Ovariectomy**	**Gabapentin Reduce Stress and Does Not Affect Ocular Parameters in Clinically Normal Cats**	**Daily Gabapentin Improved Behavior Modification Progress and Decreased Stress in Shelter Cats From Hoarding Environments in a Double-Blind Randomized Placebo-Control Clinical Trial**	**Effects of a Single Preappointment Dose of Gabapentin on Signs of Stress During Transportation and Veterinary Examination**
Authors	M. Fantinatu; J. Trnka	C. Crowe; D. Groth	H. Eagan; K. van Haaften	K. van Haaften; L. Forsythe
Year of publication	20 March	22 September	23 September	17 November
Number of cats	60	11	32	20
Dosage	5 mg/kg	10 mg/kg	10 mg/kg	100 mg/cat SID
Purpose of the study	To evaluate the appetite-stimulating effect of gabapentin by comparing it with mirtazapine in healthy cats in the first 8 h post ovariectomy.	To describe the effect of gabapentin on ocular and behavioral parameters following oral administration in healthy cats.	To evaluate the impact of daily gabapentin on behavior modification progression and signs of stress in fearful shelter cats from hoarding environments.	To determine the effects of oral gabapentin administration prior to veterinary examination on signs of stress in cats.
Outcome	Gabapentin provides a greater intake of food from the first 2 h post extubation.	Gabapentin reduces stress and increases sedation at 1.5 h after treatment, with no significant effect on horizontal pupil diameter, intraocular pressure, or Schirmer test	Gabapentin was beneficial in behavior modification progress and reduced signs of stress in shelter cats.	Gabapentin is a safe and effective treatment for cats to help reduce stress and aggression and increase compliance for transportation and veterinary examination.
Number of references	47	22	50	28
Type of article	Double-masked, placebo-controlled, prospective clinical trial	Masked, placebo-controlled, randomized crossover	Double-blind randomized placebo-controlled clinical trial	Randomized blinded, crossover clinical trial
Side effects	No side effects	None	Intermittent diarrhea	Vomiting (6 cats), hypersalivation, anisocoria, minor fasciculation
Scoring Systems	None	Stress score; sedation score; compliance score	Behavioral modification; cat stress score; latency to emerge	Aggressive scale; sedation score; cat stress score; compliance score
**Articles**	**Use of single-dose oral gabapentin to attenuate fear responses in cage-trap confined community cats: a double-blind, placebo-controlled field trial**	**Orally administered gabapentin and alprazolam induce comparable levels of anxiolysis and sedation in cats**	**Long term use of gabapentin for musculoskeletal disease and trauma in three cats**	**A comparative study between integrative practice and preappointment gabapentin on serum cortisol in cats**
Authors	E. Pankratz; K. Ferris	V. Papageorgiou; C. Vervelidis	N. Lorenz; E. Comefros	N. Versteg; T. Dias
Year of publication	17 June	24 February	13 November	24 August
Number of cats	53	60	3	20
Dosage	Low dosage: 50 mg/cat High dosage: 100 mg/cat (9.2–47.6 mg/kg)	100 mg/cat (32 mg/kg)	6.5 mg/kg BID for 5 days; and 1 month	50 mg to 1.4 to 2 kg; 75 mg to 2.1–3 kg; 100 mg to 3.1–4 kg; 125 mg to 4.1–5 kg; 150 mg 5.1–6 kg
Purpose of the study	To evaluate the safety and the efficacy of a single dose of gabapentin for attenuation of fear response in cage-trapped confined community cats.	To assess the level of anxiolysis achieved by alprazolam and gabapentin in hospitalized cats prior to elective ovariohysterectomy and evaluate the sedative effects of these agents.	To evaluate the analgesic effect of gabapentin in musculoskeletal disease.	To assess serum cortisol in cats submitted to oral gabapentin and integrative practice during clinical care.
Outcome	Both dosages reduced fear responses in confined community cats without measurable sedation over 3 h post administration vs. placebo.	Stress score was similar in cats treated with alprazolam and gabapentin.	Gabapentin is recommended for use in persistent chronic pain or neuropathic pain.	Serum cortisol was lower when cats received the treatments.
Number of references	24	32	22	22
Type of article	Double-blind placebo-controlled field trial	Prospective blinded controlled clinical trial	Case series	Randomized double-blind study
Side effects	Hypersalivation (4 cats)	None	None	None
Scoring Systems	Cat sedation score; global sedation score; facial injury score	Handling score; cat stress score; Volpato stress score	None	None
**Articles**	**Evaluation of the effects of gabapentin on the physiologic and echocardiographic variables of healthy cats: A prospective, randomized and blinded study**	**Randomized clinical trial evaluating the effect of a single preappointment dose of gabapentin on signs of stress in hyperthyroid cats**	**Hemodynamic, Echocardiographic, and Sedative Effects of Oral Gabapentin in Healthy Cats**	**Use of Gabapentin or Alprazolam in cats during postoperative, short-term hospitalization**
Authors	T. Veronezi; D. Lopes	M. Gourney; L. Gower	M. Allen; N. LeBlanc	V. Papageorgiou; C. Ververidis
Year of publication	22 September	22 February	21 December	24 June
Number of cats	40	47	10	55
Dosage	31.35 mg/kg (18.79–47.61 mg/kg)	20 mg/kg	100 mg/cat for 3–4 kg; 150 mg/cat for 4.1–7 kg (21–36 mg/kg)	100 mg/cat BID for 2 days
Purpose of the study	To evaluate, using echocardiography, the effect of a single oral administration of gabapentin on the physiologic variables and systolic and diastolic cardiac function of healthy cats.	To evaluate the efficacy of gabapentin as an anxiolytic in hyperthyroid cats.	To evaluate sedative, hemodynamic, and echocardiographic effects of cats receiving single-dose, oral gabapentin.	To assess the anxiolytic effects of gabapentin or alprazolam during short-term post operative hospitalization.
Outcome	Gabapentin improved evaluation of diastolic function of the LV and did not show adverse effects on the cardiovascular hemodynamics of young healthy cats.	Hyperthyroid cats medicated with gabapentin were more relaxed during transportation and more compliant during physical examination than cats that were administered placebo.	Single-dose oral gabapentin is well tolerated in healthy cats and produces a modest decrease in several echocardiographic parameters of systolic function; however, all affected variables remained within established reference ranges. These results suggest that gabapentin may be an appropriate sedative to administer before echocardiography in cats necessitating mild sedation.	Gabapentin reduced stress and serum cortisol; it reduced stress in cats and has a powerful analgesic impact.
Number of references	24	4	27	57
Type of article	A prospective, randomized and blinded study	Randomized clinical trial	Prospective, double-blinded, placebo-controlled, crossover study	Prospective, randomized study
Side effects	None	None	None	None
Scoring Systems	Relaxation/sedation score	None	Sedation scoring system	Demeanor handling score; cat stress score; Glasgow scale; food score intake
**Articles**	**Effect of gabapentin on ambulatory, direct, systemic arterial blood pressure in apparently healthy cats in the at-home and in-clinic environments**	**Changes in the stress markers cortisol and glucose before and during intradermal testing in cats after single administration of pre-appointment Gabapentin**	**Behavioral cardiovascular effects of a single dose of gabapentin or melatonin in cats: A randomized, double-blind, placebo-controlled trial**	**Clinical evaluation of the effects of a single oral dose of gabapentin on fear-based aggressive behaviors in cats during veterinary examinations**
Authors	M. De Lombaert, B. Lourenco	P. Hudec; C. Griffin	G. Tuleski; M. Silveira	M. Kruszka; E. Graff
Year of publication	23 April	19 January	22 August	21 December
Number of cats	6	16	75	55
Dosage	100 mg/Cat (13.8–22.6 mg/Kg)	Max. 175 mg/cat (25–35.7 mg/kg)	100 mg/cat (20 mg/Kg)	1 envelope labeled if less than 7 kg; 2 envelopes labeled if >7 kg (17–36 mg/kg)
Purpose of the study	To investigate the effects of gabapentin on direct, systolic arterial BP in cats in at-home and in-clinic environments.	To determine whether utilizing pre-appointment gabapentin will alter stress before and during intradermal testing, as determined by cortisol/glucose concentrations.	To verify whether a single dose of oral gabapentin or melatonin given 60 min before a cardiac evaluation would reduce anxiety without interfering with heart rate, systemic blood pressure, or electrocardiogram and echocardiographic index.	To investigate the effect of a single oral dose of gabapentin on fear-based aggressive behavior in cats during veterinary examinations.
Outcome	Gabapentin does not directly or indirectly affect BP.	Gabapentin did not significantly decrease cortisol/glucose concentrations. A sedative effect, rather than suppression of the pituitary-adrenocortical axis. Gabapentin would not alter intradermal test.	Gabapentin tranquilized the cats when it was given 60 min before the evaluation without interfering with systolic blood pressure and echocardiographic indexes.	Oral administration of gabapentin in cats 2 h before a veterinary visit can reduce fear-aggressive behavior during physical examination, enabling more complete evaluation.
Number of references	26	14	60	38
Type of article	Prospective, randomized, placebo-controlled, masked, crossover experimental study	Randomized, single-blinded, crossover clinical trial	Randomized, double-blind, placebo-controlled trial	Double-blind, randomized, placebo-controlled, crossover
Side effects	One case of mild ataxia	Vomiting (2 cats)	The proportion of sedated cats with Gabapentin was lower than that in the melatonin group	Vomiting (1 cat), Ataxia (3 cats), Hypersalivation (9 cats), Myorelaxation
Scoring Systems	Cat stress score; cat compliance score	Compliance score; owner assessment	Compliance score; sedation score	Compliance score and compliance score progression during the visit; ease of treatment administration
**Articles**	**Thermal antinociceptive effect of orally administered gabapentin in healthy cats**	**Effects of transdermal mirtazapine and oral gabapentin as pre-veterinary visit pharmaceuticals for shelter cats**	**Assessment of the effect of gabapentin on blood pressure in cats with and without chronic kidney disease**	**Assessment of the effects of gabapentin on activity levels and owner-perceived mobility impairment and quality of life in osteoarthritic geriatric cats**
Authors	Bruno H. Pypendop, Kristine T. Siao et al.	Vanessa Spano, Cary M. Springer et al.	Jessica M Quimby 1, Sarah E Jones et al.	Alonso G P Guedes, Julie M Meadows et al.
Year of Publication	October 2010	June 2023	May 2024	September 2018
Number of cats	6 cats	94 cats	29 cats	20 cats
Dosage	5; 10 or 30 mg/kg	100 or 200 mg/cat	10 mg/kg	10 mg/kg
Purpose of the study	To determine the thermal antinociceptive effect of various single doses of gabapentin administered orally in cats.	To evaluate the effect of transdermal mirtazapine on the amount of gabapentin-laced food ingested, and the effect of gabapentin on signs of fear and anxiety in cats when handled for examination.	To assess the effect of gabapentin on blood pressure (BP) in cats with and without chronic kidney disease.	To evaluate effects of gabapentin on activity levels and owner-perceived mobility impairment and quality of life (QOL) in osteoarthritic geriatric cats.
Outcome	Orally administered gabapentin did not affect the thermal threshold in healthy cats and therefore did not appear to provide thermal antinociception.	Pre-treatment with transdermal mirtazapine increased cats’ consumption of food laced with gabapentin in powder form; its stimulating effects may have counteracted the psychoactive effects of gabapentin.	Gabapentin may decrease arterial BP in cats with and without CKD and these findings should be taken into account when gabapentin is administered to patients in which measurement of BP is needed.	Gabapentin treatment was associated with significant improvements in owner-assessed QOL, mobility impairment, and pain behavior.
Number of references	31	45	27	30
Type of Article	Clinical study	Double-blinded, placebo-controlled study	Randomized, blinded, placebo-controlled crossover study	Clinical study
Side effects	None	None	None	None
Scoring System	Thermal threshold testing system	Global sedation score	Compliance score; sedation score	Compliance score; activity level; quality of life score

**Table 2:** Risk of bias assessment for the 20 included studies.

Study	Study Design	Risk of Bias Tool	Overall Risk	Notes
Pypendop et al. (2010) [23]	Experimental (pain threshold)	ROBINS-I	Moderate	No control group; outcome measurement blinded
Kruszka et al. (2021) [24]	Observational	ROBINS-I	Serious	No comparator group, subjectivity in behavioral outcomes
Pankratz et al. (2017) [25]	RCT	SYRCLE	Low	Good randomization, blinding, placebo group
Versteg et al. (2024) [26]	Controlled observational	ROBINS-I	Moderate	Parallel group, some confounding risk
Allen et al. (2021) [27]	RCT	SYRCLE	Low	Well-designed physiological study
Lorenz et al. (2013) [28]	Case series	ROBINS-I	Serious	No controls, retrospective description
van Haaften et al. (2017) [29]	RCT	SYRCLE	Low	Pre-appointment stress assessment, well controlled
Tuleski et al. (2022) [30]	RCT	SYRCLE	Moderate	Small sample, limited blinding of outcome assessors
Veronezi et al. (2022) [31]	RCT	SYRCLE	Low	Blinded echocardiographic assessment
De Lombaert et al. (2023) [32]	Observational	ROBINS-I	Moderate	BP assessment, no randomization
Quimby et al. (2024) [33]	Observational	ROBINS-I	Moderate	CKD cats vs. healthy, no blinding
Gurney and Gower (2022) [34]	RCT	SYRCLE	Low	Randomized, controlled in hyperthyroid cats
Crowe et al. (2022) [35]	Observational	ROBINS-I	Moderate	Objective ocular outcomes, but unblinded
Hudec et al. (2019) [36]	Observational	ROBINS-I	Serious	Cortisol assessment, invasive procedures bias
Guedes et al. (2018) [37]	Controlled trial	ROBINS-I	Moderate	Placebo-controlled, some self-report limitations
Eagan et al. (2023) [38]	RCT	SYRCLE	Moderate	Randomized, but inconsistent outcome reporting
Papageorgiou et al. (2024) [39]	Controlled (non-randomized)	ROBINS-I	Serious	Compared to alprazolam, not randomized
Spano et al. (2023) [40]	Observational (cross-over)	ROBINS-I	Moderate	Compared gabapentin to mirtazapine in shelter cats
Fantinatu et al. (2020) [9]	RCT	SYRCLE	Low	Appetite-focused, placebo-controlled
Steagall and Monteiro (2019) [41]	Review	Not applicable	Not rated	Not original data (excluded from RoB table)

## Data Availability

No new data were created or analyzed in this study.
